# Long-term metapopulation study of the Glanville fritillary butterfly (*Melitaea cinxia*): survey methods, data management, and long-term population trends

**DOI:** 10.1002/ece3.733

**Published:** 2013-09-08

**Authors:** Sami P Ojanen, Marko Nieminen, Evgeniy Meyke, Juha Pöyry, Ilkka Hanski

**Affiliations:** 1 Department of Biosciences, University of HelsinkiPO Box 65 (Viikinkaari 1), FI-00014, Helsinki, Finland; 2 Natural Environment Centre, Finnish Environment InstituteP.O. Box 140, FI–00251, Helsinki, Finland

**Keywords:** Climate change, EarthCape, Glanville fritillary, habitat fragmentation, long-term population survey, management of ecological data, spatial synchrony of population dynamics

## Abstract

Long-term observational studies conducted at large (regional) spatial scales contribute to better understanding of landscape effects on population and evolutionary dynamics, including the conditions that affect long-term viability of species, but large-scale studies are expensive and logistically challenging to keep running for a long time. Here, we describe the long-term metapopulation study of the Glanville fritillary butterfly (*Melitaea cinxia*) that has been conducted since 1991 in a large network of 4000 habitat patches (dry meadows) within a study area of 50 by 70 km in the Åland Islands in Finland. We explain how the landscape structure has been described, including definition, delimitation, and mapping of the habitat patches; methods of field survey, including the logistics, cost, and reliability of the survey; and data management using the EarthCape biodiversity platform. We describe the long-term metapopulation dynamics of the Glanville fritillary based on the survey. There has been no long-term change in the overall size of the metapopulation, but the level of spatial synchrony and hence the amplitude of fluctuations in year-to-year metapopulation dynamics have increased over the years, possibly due to increasing frequency of exceptional weather conditions. We discuss the added value of large-scale and long-term population studies, but also emphasize the need to integrate more targeted experimental studies in the context of long-term observational studies. For instance, in the case of the Glanville fritillary project, the long-term study has produced an opportunity to sample individuals for experiments from local populations with a known demographic history. These studies have demonstrated striking differences in dispersal rate and other life-history traits of individuals from newly established local populations (the offspring of colonizers) versus individuals from old, established local populations. The long-term observational study has stimulated the development of metapopulation models and provided an opportunity to test model predictions. This combination of empirical studies and modeling has facilitated the study of key phenomena in spatial dynamics, such as extinction threshold and extinction debt.

## Introduction

Ecological studies of local populations and population processes tend to last for a few years only and typically encompass a small spatial scale (Kareiva and Andersen 1988). At very large spatial scales, there are long-term monitoring programs, such as the Rothamsted Insect Survey, which has sampled moths and aphids at tens of permanent sites across the U.K. for nearly 50 years (Taylor 1986; Woiwod and Hanski 1992; Conrad et al. 2002), and the PISCO project, a long-term study of marine populations, communities, and ecosystem processes along the west coast of the United States (Broitman et al. 2008; Barshis et al. 2011; Menge 2012). However, in such projects the actual, fine-scale spatial structure of populations remains poorly known and only a few populations can be sampled, which leaves many questions about spatial population processes unstudied.

Metapopulation studies extend the traditional population ecological studies to larger spatial scales, to multiple interacting populations, and to the processes underpinning spatial dynamics (Hanski 1999; Hanski and Gaggiotti 2004b). Metapopulation studies typically aim at covering networks of local populations at spatial scales that are at least of the same order of magnitude in size than the average dispersal distance of the focal species. Such regional-scale studies that are conducted on well-defined assemblages of local populations make several contributions to ecology, population biology, and conservation biology. First, landscape (habitat) structure and context are likely to greatly influence population dynamics (Fahrig 1988; Ovaskainen and Hanski 2004), life-history ecology (Ronce and Olivieri 2004), and evolutionary dynamics (Whitlock 2004; see also other chapters in Hanski and Gaggiotti 2004a). To be informative, empirical studies of landscape effects have to be conducted in large heterogeneous regions. For instance, a good understanding of landscape effects is needed for a mechanistic understanding of the biological consequences of habitat loss and fragmentation, which are the major causes of declining biodiversity worldwide (Hanski 2005; Tscharntke et al. 2005; Fahrig et al. 2011).

Second, the demographic and microevolutionary dynamics of populations are often strongly influenced by dispersal and gene flow among populations, which is evident in the case of source–sink populations: the presence, ecological dynamics, genotypic composition, local adaptation, and so forth of sink populations depend critically on the surrounding populations (Kawecki 2004, 2008). There is presently much interest in coupled demographic and microevolutionary dynamics (eco-evolutionary dynamics; Pelletier et al. 2009; Schoener 2011), which is likely to be especially important in metapopulations inhabiting heterogeneous environments (Hanski 2011, 2012b).

Third, in highly fragmented landscapes consisting of many small habitat patches, local populations are not likely to persist for a long time because of their generally small size, and hence long-term persistence and practically anything else related to the biology of the species depend on metapopulation-level processes and hence call for metapopulation-level studies. Here, key questions relate to the rate and causes of population turnover and the degree of spatial synchrony in population dynamics (Hanski 1999).

**Figure 1:**
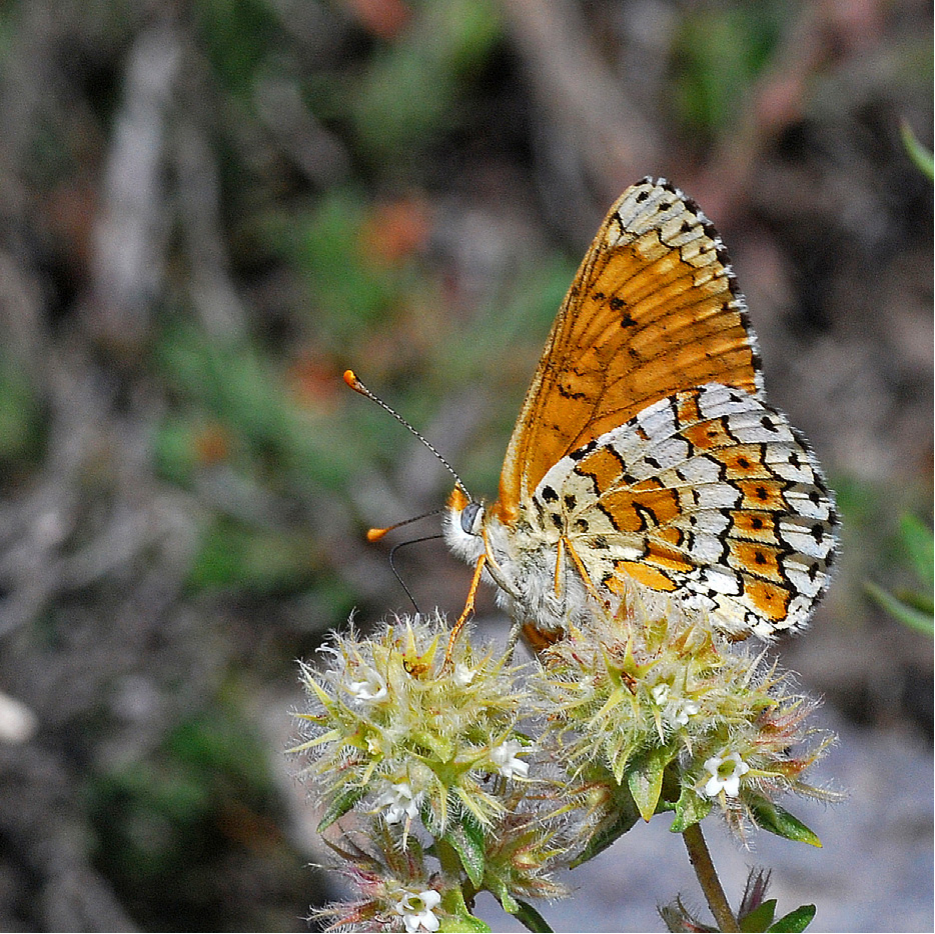
The Glanville fritillary butterfly (*Melitaea cinxia*). Photograph courtesy of Hannu Aarnio.

Studies that have continued for many generations allow researchers to investigate population trends and other patterns in population fluctuations. These questions have become especially topical in the context of climate change (Parmesan 1996; Parmesan et al. 1999). Long-term studies are necessary to develop a mechanistic understanding of the role of the demographic, genetic, and microevolutionary processes that influence population dynamics, as well as of the spatial and temporal scales at which these processes are likely to occur. Spatial variation in landscape structure can often be substituted for variation in time to study the likely consequences of changing landscape structure, but ideally one would like to continue a large-scale study long enough to examine the actual temporal changes. The reasons why there are not many long-term population studies at large spatial scales include the cost of such research, the need to establish research infrastructure for the long-term study, and various other logistic difficulties in working at large spatial scales. Notable examples of long-term metapopulation projects include studies on small mammals (reviewed by Lambin et al. 2004), butterflies (reviewed by Thomas and Hanski 2004), and plants (reviewed by Antonovics 2004). Here, our purpose is not to review long-term metapopulation studies, but to provide a benchmark for such studies by describing the very large-scale and long-term study of the Glanville fritillary butterfly (*Melitaea cinxia*) in Finland (Fig. 1). This study was started in 1991, and it was expanded to its current large spatial scale in 1993, covering a network of 4000 discrete habitat patches (dry meadows) and the respective local populations within an area of 50 by 70 km (Hanski 1999, 2011; Nieminen et al. 2004). The annual metapopulation survey is integrated into a range of targeted ecological, genetic, and evolutionary studies. We explain here the description of the landscape structure and the habitat of the species, logistics, cost, and reliability of the metapopulation survey, data management, and the major long-term trends in the dynamics as revealed by the survey.

## The Glanville Fritillary Butterfly

**Figure 2:**
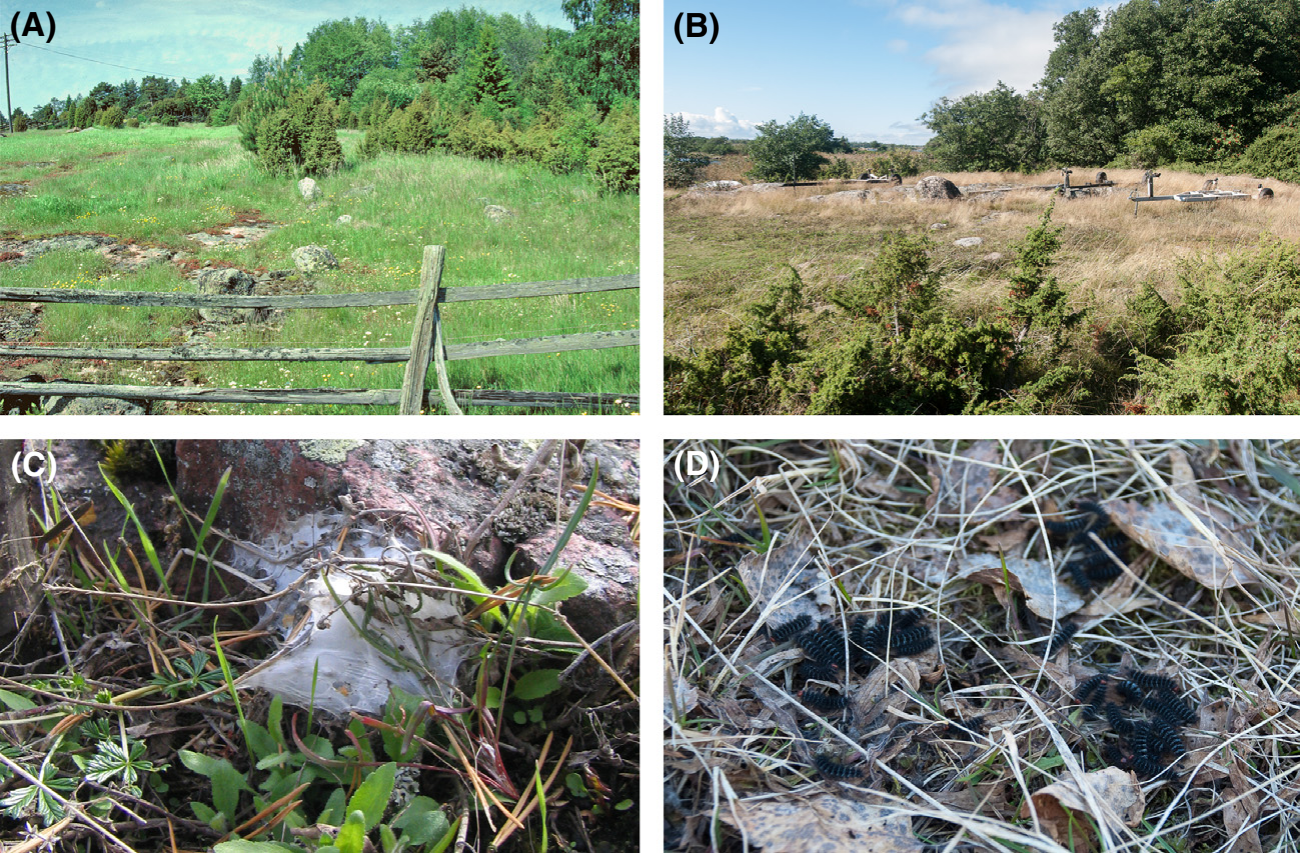
(A) and (B) show representative examples of dry meadows used by the Glanville fritillary as breeding habitat; (C) a “winter nest” in early September, inside which a group of full-sib larvae diapause; and (D) postdiapause larvae basking in small groups in April.

The Glanville fritillary butterfly (*M. cinxia* L.) has one generation per year in northern Europe, adults flying from June to early July. In the Åland Islands in south-west Finland, females lay eggs in clusters of 50–250 (mostly 150–200) eggs on two host plant species, *Plantago lanceolata* L. and *Veronica spicata* L. (Kuussaari 1998; Nieminen et al. 2004). Larvae hatch in 2–3 weeks, forage gregariously and spin a web around the host plant, in which they stay at night, during bad weather and when not feeding. Half-grown larvae overwinter in compact “winter nests”, which they spin at the base of the host plant at the end of August (Fig. 2C). The larvae resume feeding in the spring when host plants start to grow, usually in the beginning of April, remaining gregarious until the final instar. Pupation takes place in May. Further details of the life cycle and life history are reported by Kuussaari (1998); Nieminen et al. (2004); Hanski (1999); Hanski et al. (2006); Saastamoinen (2007); and Saastamoinen et al. (2009).

The fact that each larval group spins a winter nest (Fig. 2C) before winter diapause makes the large-scale survey of local populations possible. The winter nests are conspicuous in early September, and it is feasible to aim at counting all the winter nests on every meadow in a network of thousands of meadows, giving an estimate of local population sizes across the entire study area as well as an opportunity to sample larval family groups for experiments. Since 1991, a large number of specific studies have been conducted on the behavior, ecology, genetics, and evolution of the Glanville fritillary (Table 1). The transcriptome was described by Vera et al. (2008a) and the full genome will be published in 2013.

**Table 1:** A selection of behavioral, ecological, genetic, and evolutionary studies on the Glanville fritillary

Subject	Selected references
Mating behavior	Haikola et al. (2004)
Oviposition host plant preference and its evolution	Kuussaari et al. (2000); Saastamoinen (2007); Saastamoinen and Hanski (2008)
Movement behavior	Kuussaari et al. (1996); Hanski et al. (2000, 2006); Saastamoinen (2008)
Larval behavior and biology	Kuussaari et al. (2004)
Inbreeding and its demographic consequences	Saccheri et al. (1998); Haikola et al. (2001); Nieminen et al. (2001)
Genetic effects on life-history traits	Orsini et al. (2009); Saastamoinen et al. (2009)
Local population dynamics	Kuussaari et al. (1998)
Genetic causes of population dynamics	Hanski and Saccheri (2006)
Metapopulation dynamics	Hanski et al. (1995, 1996); Hanski and Ovaskainen (2000)
Spatial genetic structure	Orsini et al. (2008)
Eco-evolutionary metapopulation dynamics	Hanski (2011); Hanski et al. (2011)
Evolution of dispersal rate	Heino and Hanski (2001); Zheng et al. (2009); Hanski and Mononen (2011)

## Description of the Study Landscape

**Figure 3:**
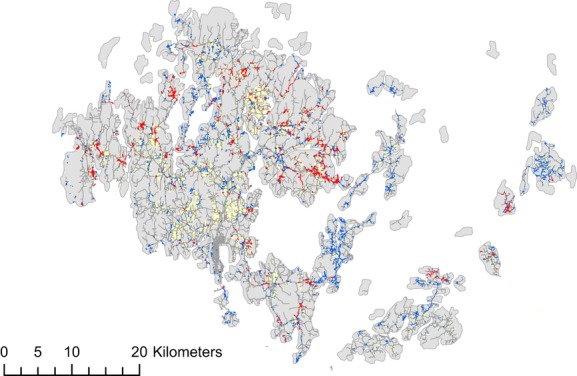
The Åland Islands, showing the spatial locations of ca. 4000 meadows. Meadows occupied in 2012 are shown with red and unoccupied meadows with blue. Cultivated fields are shown with yellow color and roads with gray lines.

The Åland Islands consists of the main island of 685 km^2^, several inhabited medium-sized islands from 5 km^2^ to 85 km^2^, and a very large number of small islands and islets (Fig. 3). Most of the small islands lack suitable habitat for the Glanville fritillary and are hence not relevant in the present context. The landscape is heterogeneous. On the main island, the main land-use types are agricultural land (cultivated fields, pastures), managed mixed forests, largely unmanaged rocky areas (open pine-dominated areas), and built areas (a small town, villages, isolated houses, and summer cottages).

The larval host plants, *P. lanceolata* and *V. spicata,* grow on dry meadows, pastures, and comparable habitats, which occur mostly as well-defined, discrete habitat patches (Fig. 2A, B). The key criterion of breeding habitat is the presence of at least one of the two host plant species. The larvae feed gregariously in groups that have initially 50–250 larvae (Kuussaari 1998; Kuussaari et al. 2004). Individual host plants are so small that a large larval group will defoliate the entire plant individual on which the female oviposited the egg cluster, and hence the larval group has to move to another nearby plant. Therefore, if there are very few host plants and they are very scattered, the site may not allow successful development of even a single larval group, especially in years in which many plants dry out (below).

We have systematically and thoroughly mapped the habitat patches in the study area using topographic maps and by visiting all potentially favorable areas. This task has been facilitated by the 908 km (in 1995) of paved and unpaved public roads and roughly 2500 km of narrow unpaved farm roads. Details of habitat mapping are described in the Appendix. The current number of habitat patches is 4248 (in 2012) with the pooled area of 783 ha, which covers 0.5% of the total land area (1 552 km^2^).

The area of each habitat patch is a key parameter, as patch area has played a critical role in the development of metapopulation models for the Glanville fritillary (Hanski 1994; Hanski et al. 1996, 2011; Hanski and Ovaskainen 2000; Ovaskainen and Hanski 2004). To make the delimitation of habitat patches as consistent as possible, the patches have been delimited by only three field assistants (FA) using a Global Positioning System (GPS) receiver (Corvallis Microtechnology Inc., Corvallis, OR). Inevitably, there are a number of complications, which are described in detail in the Appendix.

## Data Management: the EarthCape Biodiversity Platform

In the early years of the survey, we had sets of topographic maps on which the habitat patches had been drawn, and paper forms, one for each patch, on which data were recorded. Since 2010, we have implemented a comprehensive database management system, into which we have integrated data collection in the field as well as subsequent tasks related to the management of larval samples collected during the survey (below) and various tasks related to data analysis. We use EarthCape database management system (http://www.earthcape.com), consisting of a set of desktop and web server database applications specifically designed for biodiversity data collection, management, analysis, and publication. EarthCape is also used to streamline the planning of the metapopulation surveys, recording of the data in the field and in the laboratory, and it is used in data exploration and visualization. A brief description of the functions of EarthCape has been presented in the Appendix.

**Figure 4:**
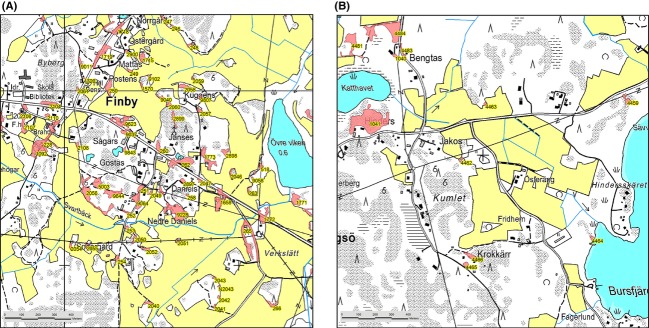
Two examples of GPS-delimited habitat patches. (A) Shows an area where patch density is high and (B) an area where the network is sparse. The difference mostly relates to the openness of the landscape, which typically depends on human land use but also on, for example, soil type and thickness (many meadows occur on rocky outcrops with little soil).

Before each annual metapopulation survey (below), we extract data from the main database to plan the amount of resources needed for field work. Map files and current patch outlines (Fig. 4) are transferred to notebook computers with a customized copy of the database. Since 2010, we have used digital, zoomable maps of the habitat patches in small notebook computers (Lenovo S10-2, Morrisville, NC, and Samsung models NC110, N210, and NF310, Seoul, South Korea) that are connected to a GPS device (Transystem iBlue 737A+ and 747A+ GPS receivers with AGPS function, Hsinchu, Taiwan) via Bluetooth. Barcode stickers are printed out to be used for larval samples collected in the field. The same physical stickers move further down the pipeline with larvae in the laboratory, where larvae are reared and phenotyped following the winter diapause.

## Metapopulation Survey

### The logistics of the survey

The annual survey is organized from late August to early September, at the time when the larvae have woven the winter nest that is relatively easy to find in the field (Fig. 2C; see Video S1). The field work is done by pairs of FAs. Prior to the survey, the FAs attend an orientation lecture, and survey-related concepts and tasks are demonstrated in practice in the first day in the field, including searching for larval groups and recording of data on habitat patches and host plants. Each group of FAs has a car, and they end up driving 50–100 km per day while visiting and surveying on average 20 habitat patches per day.

The details of the field work are described in the Appendix. Briefly, the amount of time spent searching for larval groups in each patch is proportional to patch area. If no larval groups are detected within the prescribed search time, the entire patch is re-searched using the same search time to reduce the number of false negatives (this is important for patch occupancy metapopulation models; Ovaskainen and Hanski 2004). In the spring, the habitat patches in which larvae were found in the previous autumn are searched for postdiapause larvae (Fig. 2D). In the spring, the number of individual larvae in each group is counted. For instance, 137,000 larvae were counted in the spring 2012, which was a record year (Fig. 6 below). Additionally, the numbers of hatched and nonhatched cocoons of the primary parasitoid *Cotesia melitaearum* (Wilkinson) are counted in each larval group (for description of the parasitoid assemblage see Lei and Hanski 1997; Lei et al. 1997; van Nouhuys and Hanski 2004).

The total cost of the autumn survey is around €150,000, whereas the spring survey is cheaper, typically €15,000–20,000, because only the populations that existed in the previous autumn are visited (Table 2). Details of the costs are described in the Appendix.

**Table 2:** The cost of the Glanville fritillary monitoring in 2010

	Autumn 2010	Spring 2010
Number of field assistants	72^1^	17^2^
Duration of the field work	15 days	6 days
Cost item (€)		
Salaries and related costs per field assistant	1,600	780
Travel costs^3^	14,100	4,700
Accommodation	10,700	830
Computers, etc.	6,100	450
Total	150,100	22,700

The total cost includes the salary for the director of field work as well as various miscellaneous costs in addition to the costs specified in the table.

1 Includes 70 assistants, the director, and his deputy.

2 Includes 16 assistants and the director.

3 Includes allowances for the use of own cars.

### Recording of the data in the field

Recording of the data in the field has been under constant changes since 1993 due to development in computer hardware. Since 2010, all data have been recorded directly on small rain-protected notebook computers, which contain a local copy of the master database. The computers have the topographic map of the Åland Islands to help the FAs orient themselves to the next habitat patch. When on the spot, FAs may display the patch outline in the geographic information system (GIS) viewer on top of a detailed topographic map. All patch-specific information is available, such as the records of larval groups, past information on host plants, and so forth. The survey coordinator collects the data from each field computer into a single database every evening to construct preliminary pivot tables that enable spotting missing data, obvious outliers, areas still to be surveyed, and so forth. The ability to see and explore the data on the map and the easy preview of the data in, for example, Google Earth make a big difference when cleaning up the data (see Video S2). Data are synchronized using EarthCape import/export mechanism, which also serves a backup purpose. Details of the data recorded during the survey are described in the Appendix.

### Weather data

Weather conditions play an important role in the dynamics of the Glanville fritillary (Nieminen et al. 2004; Hanski and Meyke 2005). Precipitation data have been obtained from weather radar since 1998. The spatial and temporal resolutions of these data are 0.5 × 0.5 km and 5 min, respectively. In addition, we have placed portable temperature and humidity data loggers (Lascar Electronics, EL-USB2, Salisbury, U.K.) in 50 representative habitat patches since 2009. The loggers are placed in the field in early April and recovered during the fall survey in August to September. The loggers are mounted about 30 cm above the ground and shaded from direct sunlight with a white plastic half-dome cover. A separate black button recorder (Maxim iButton DS1922L, Sunnuvale, CA) is planted in a subset of the sites to measure temperatures that basking larvae are able to reach in the spring.

### Sampling of populations

In 1995, 2002, and every year since 2007, a sample of two or three larvae has been taken from every larval group detected in the field for phenotypic and genotypic measurements. Due care is taken to keep the level of disturbance as low as possible (see Appendix). Information on the larval sample is entered into the database at the time of sampling. The tubes with larvae are labeled with preprinted barcode labels with appropriate information and stored in a cool dark place until transferred to the laboratory. Labels are read into the database in the field. Using barcodes reduces errors in the labeling of samples, and reading barcodes saves time, which is an important consideration while dealing with thousands of samples.

### Reliability of the survey

Given the size of the study area (50 by 70 km) and the large number of discrete habitat patches (4000 meadows), it is clear that the survey of population sizes cannot be exhaustive. Several approaches have been used to estimate the probability of detecting a larval group during the autumn survey. In 1994, 1995, and 1997, intensive surveys of four habitat patches (different patches in each year) were conducted to obtain a value for the “true” number of larval groups, after which eight independent pairs of FAs conducted the survey with the usual search effort. In 2008, 67 patches were surveyed twice, with the same search effort in each survey, this time with the second pair of FAs knowing the nest count from the first survey. Using a Bayesian model to analyze these data sets, Harrison et al. (2011) estimated that 50% of the existing larval groups were found during the first search. In 2009 and 2011, 180 and 80 habitat patches were resurveyed, respectively, by a large number of FAs spending much time in each patch. Altogether 1304 larval groups were found, 809 (62%) of which were detected during the first search. Assuming that almost all larval groups were found during the thorough re-search, we conclude that the probability of detecting a larval group is 0.5–0.6 in the regular survey. This result has been incorporated into modeling of metapopulation dynamics (Harrison et al. (2011).

When the patch is judged to be unoccupied during the regular search, it is immediately re-searched with the same effort. In the controls done in 2009 and 2011, 72% of the meadows considered to be unoccupied turned out to be unoccupied also after the second search. Of the remaining 28%, half had only one or two larval groups and the rest had >2 larval groups based on the second search. The most common reason for missing larger numbers of larval groups in the regular search was that a part of the patch had not been searched at all for some reason, for instance, because the patch boundaries were misinterpreted. This problem has been largely eliminated in recent years by having the outline of the patch displayed on an accurate topographic map in the field computer.

## Long-Term Metapopulation Dynamics of the Glanville Fritillary

The habitat patch network in the Åland Islands has remained relatively stable since 1993. Previously recognized patches have disappeared mainly due to overgrowth by grasses and bushes in the absence of grazing and other forms of disturbance, and due to construction of roads and buildings, tillage, and reforestation. Altogether ca. 550 habitat patches have thereby disappeared in 1994–2011, which makes 30 patches per year, or roughly 1% per year.

**Figure 5:**
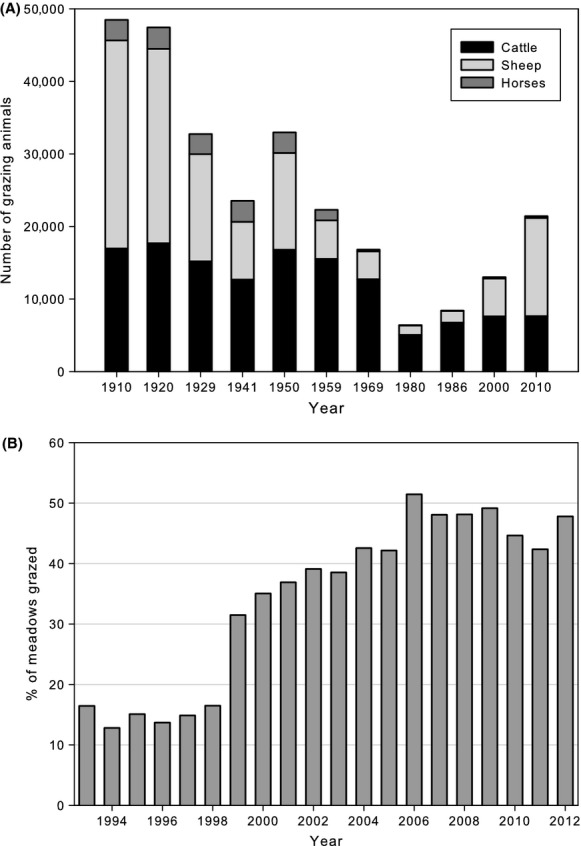
(A) Changes in the number of grazing animals in the Åland Islands since 1910 (Source: Finnish Information Centre of the Ministry of Agriculture and Forestry and the preceding agencies) and (B) the percentage of meadows in the Glanville fritillary study system that have been grazed by domestic mammals (mostly sheep and cattle).

Grazing is a key factor influencing the quality of meadows for the Glanville fritillary and many other species inhabiting similar dry meadows. In the long-term, over the past 100 years, the number of cattle, sheep, and horses declined steadily to only about 15% by 1980 (Fig. 5A). However, in the past 30 years the trend has been reversed, and the number of grazing animals has again increased to about 45% of the number in 1910, especially due to increasing number of sheep (Fig. 5A). The changes that have taken place in the past decades are reflected in the fraction of meadows with grazing animals, which has increased from about 15% in the 1990s to more than 40% in recent years (Fig. 5B).

**Figure 6:**
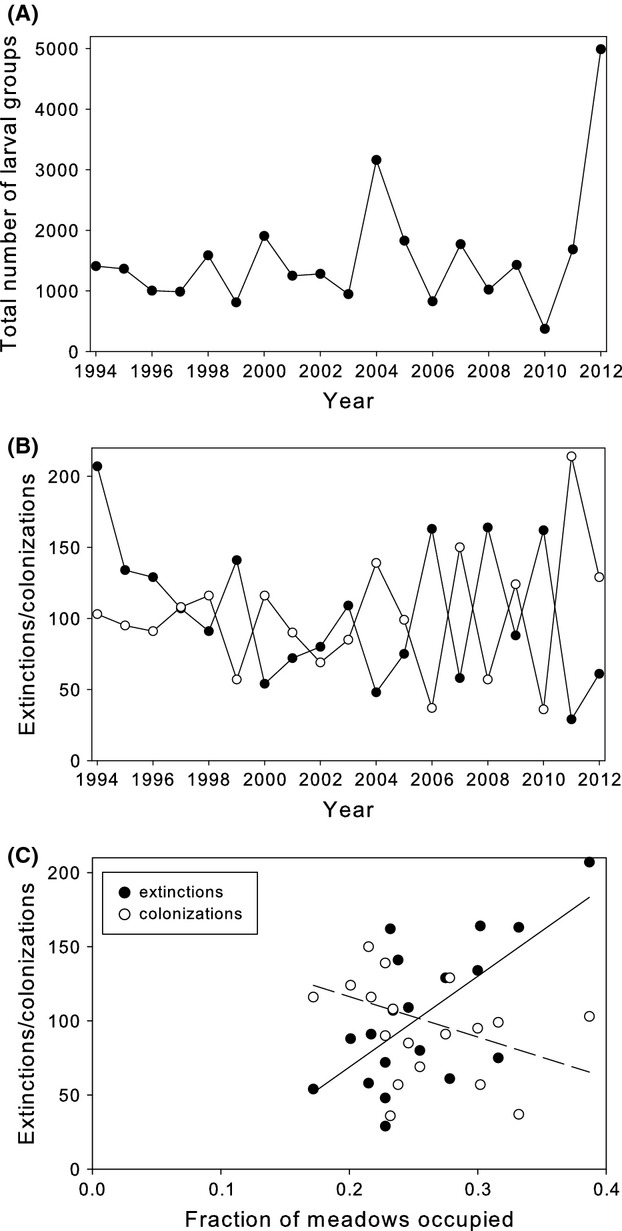
(A) The size of the Glanville fritillary metapopulation in terms of the total number of larval groups in the autumn and (B) the numbers of local extinction and recolonization events per year. The results were calculated for the meadows that have been monitored in every year during 1993–2012. (C) The numbers of annual extinction and recolonization events plotted against the fraction of meadows occupied during 1993–2012. Updated from the figure in Hanski (2011).

The size of the metapopulation in terms of the pooled number of larval groups and the number of local populations shows no long-term trend, although naturally there has been variation from one year to another (Fig. 6A). The reason for the all-time high in metapopulation size in 2012 was two consecutive favorable years for larval growth during the summer (Fig. 6A). It is noteworthy that the greatly increased fraction of grazed meadows (Fig. 5B) has had no obvious influence on metapopulation size, reflecting the fact that many of the currently grazed meadows would remain habitable, at least for some time, even without grazing. Extinction and recolonization events are frequent, roughly between 50 and 150 events per year (Fig. 6B). The numbers of annual extinctions and recolonizations depend strongly on the current number of local populations: Extinctions are more common in years when there are many local populations and vice versa for recolonizations (Fig. 6C). The two relationships intersect at a point when around 24% of the habitat patches are occupied, which thereby represents the stable state for the metapopulation.

The dynamics of insect populations are much affected by the prevailing environmental conditions, and the Glanville fritillary is not an exception. We run stepwise linear regression models to explain the annual rates of extinction and colonization with the number of local populations in the previous year (as in Fig. 6C) as well as with monthly average temperatures and precipitation. This analysis shows that recolonization rate increases, and extinction rate decreases, with increasing precipitation in July (Table 3). The reason for these effects is host plants withering in dry summers, which increases larval mortality (Nieminen et al. 2004; Hanski and Meyke 2005).

**Table 3:** Stepwise logistic regression models for the numbers of annual recolonization and extinction events

	Recolonization events			Extinction events		
		
Variable	Coeff	*t*	*P*	Coeff	*t*	*P*
Fraction occupied	−9.86	−0.07	0.94	468	3.03	0.009
Log July precipitation	58.0	3.02	0.009	−47.8	−2.2	0.045
*R*^*2*^	0.38			0.59		

The independent variables include monthly average temperatures from April to August, the logarithm of monthly precipitation in June, July, and August, and the fraction of occupied habitat patches out of all patches in the previous year (as in Fig. 6C). The fraction of occupied patches was included in the model, whereas of the remaining variables only rainfall in July had a significant effect.

**Figure 7:**
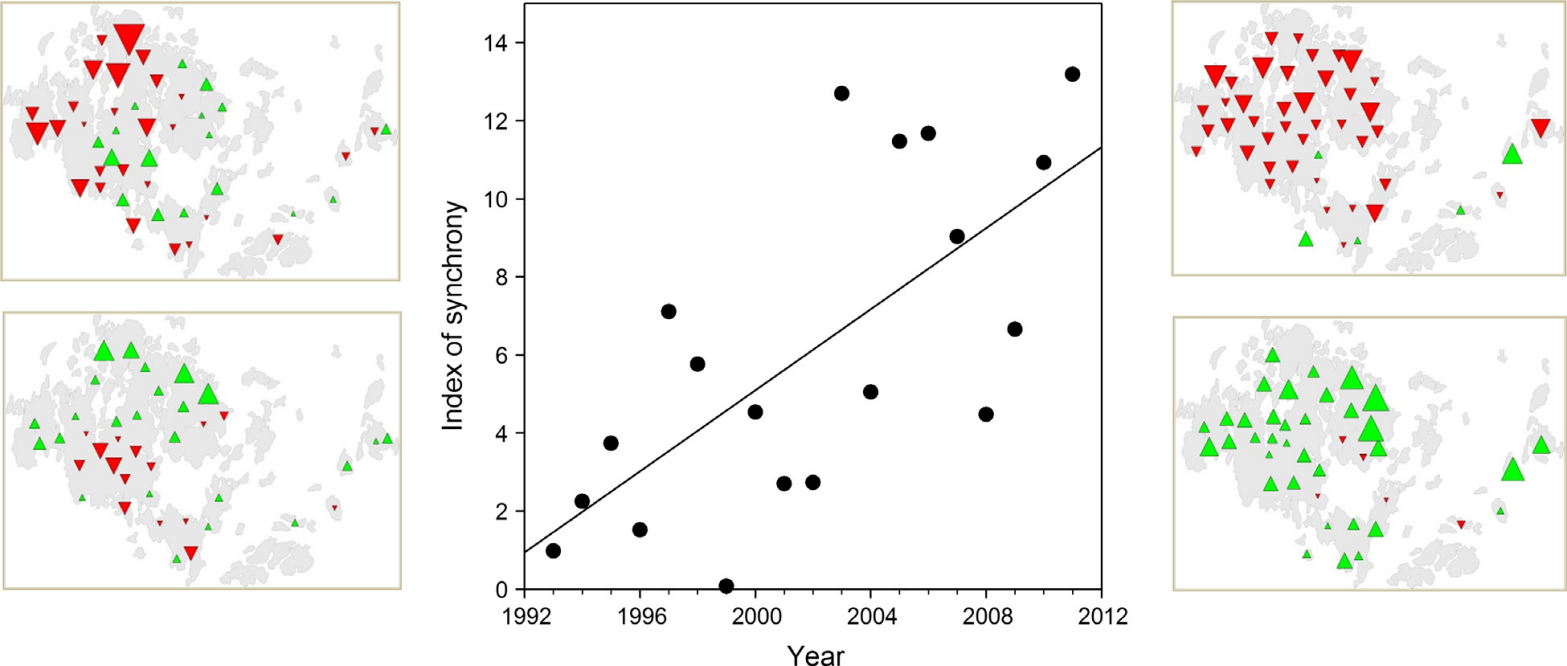
Large-scale spatial synchrony in the Glanville fritillary metapopulation during 1993–2012. The small maps on the left illustrate regional per capita changes in population sizes (log (*N*_*t+1*_/*N*_*t*_)) in the early years of the survey, during 1993–1994 (the upper map) and 1994–1995 (the lower map). Red down-pointing triangles are regions in which populations declined, green up-pointing triangles are regions in which populations increased. The size of the symbol is proportional to the magnitude of per capita change. The maps on the right give similar information for the later years of the survey, during 2005–2006 and 2006–2007. The figure in the middle shows the value of an index of synchrony against time. The index of synchrony was calculated by summing up the per capita changes shown in the small maps, with red symbols (declining populations) having a minus sign. The vertical axis of the middle figure shows the value of this sum without the sign. Thus, when the positive and negative regional changes compensate each other, as in the maps on the left, the value of the index is small. Least squares regression for the index of synchrony against year is highly significant (*P* = 0.0006, *R*^*2*^ = 0.48).

Although there is no long-term trend in the size of the metapopulation (Fig. 6A), there has been a striking change in the spatial scale of synchrony in year-to-year population dynamics. In the 1990s, the spatial scale of autocorrelation was roughly 10 km, and thus populations in different parts of the study area often changed in the opposite directions (Fig. 7). In contrast, in the past 10 years, the changes have been much more synchronous across the Åland Islands (Fig. 7), which leads to years when either recolonization greatly exceeds extinctions or vice versa (Fig. 6B). As a result, the degree of large-scale spatial synchrony has increased significantly over the years (Fig. 7). We do not know the reason for this change, but one possibility is climate change and increasing frequency of extreme weather conditions in the recent past. For instance, the very low size of the metapopulation in 2010 was largely due to record-high temperatures in July and widespread withering of the host plants leading to starvation of caterpillars. In contrast, populations have greatly increased in 2011 and 2012, when conditions for plant growth and larval development were favorable due to sufficiently high rainfall. The average July temperature in the Åland Islands has increased by ca. 1 degree in the period from 1993 to 2010.

## Discussion

### The added value of large-scale and long-term population studies

The value of long-term ecological studies is widely recognized, as such studies contribute to empirical knowledge of the dynamics of natural populations under the prevailing and possibly changing environmental conditions. Without long-term studies, we would be ignorant about the population ecological consequences of climate change and land-use changes, although clearly one has to be careful while drawing inferences from observational studies, regardless of whether they are short-term or long-term studies. An excellent example of the value of long-term population studies is the Living Planet Index (Grooten 2012), which quantifies trends in population sizes for vertebrate species from different parts of the world, based on data from more than 9000 wildlife monitoring schemes. Mere time series of population sizes are generally not sufficient to demonstrate which particular mechanisms have caused the observed changes. Nonetheless, a long-term study provides essential context for more targeted studies, and the long-term study may provide invaluable material for experiments. In the case of the Glanville fritillary, studies on the movement behavior and dispersal, and how genetic polymorphism affects mobility and other life-history traits (references in Table 1), have greatly benefitted of the knowledge about the entire metapopulation for a prolonged period of time. The results of these studies, combined with information about the spatial distribution of habitat patches in the study area, have facilitated the analysis of key phenomena in spatial population dynamics, such as extinction threshold (Hanski and Ovaskainen 2000) and extinction debt (Hanski et al. 1996). Using the large amount of life-history data has made it possible to construct predictive models of dispersal ecology and evolution (Heino and Hanski 2001; Zheng et al. 2009; Hanski and Mononen 2011) that take into account the spatial configuration of the habitat patch network.

Second, the long-term record for the large assemblage of local populations in the heterogeneous patch network has allowed comparisons between populations with different demographic histories. Thus, we have shown that small isolated populations tend to be so inbred that their risk of extinction is elevated (Saccheri et al. 1998; Nieminen et al. 2001). We have sampled larvae from newly established versus old local populations, and have shown that there are systematic differences between such populations in their genotypic (Haag et al. 2005; Hanski 2012a; Wheat et al. 2011) and phenotypic composition (Hanski et al. 2006). In particular, females from newly established populations are more dispersive than females from old local populations (Hanski et al. 2002; Ovaskainen et al. 2008), supporting the model predictions that natural selection favors more dispersive individuals in highly fragmented landscapes (Ronce and Olivieri 2004; Hanski and Mononen 2011). With these and other studies, reviewed by Hanski (1999, 2011, 2012a), and many chapters in Ehrlich and Hanski (2004), the Glanville fritillary study system has become a well-recognized model system in metapopulation biology.

The success of the Glanville fritillary project is based on integration of different types of research around a common set of questions about spatial dynamics and the consequences of habitat fragmentation. From the very beginning, empirical studies have stimulated and informed modeling studies (Hanski 1994; Ovaskainen and Hanski 2001), and many model predictions have been effectively tested with empirical data (Hanski et al. 1995, 1996; Wahlberg et al. 1996; Hanski and Ovaskainen 2000). As described above, targeted experimental studies on a range of questions have been conducted along with the long-term observational study, combination of which has helped generate funding for the long-term study. Genetic (Saccheri et al. 1998; Orsini et al. 2008) and microevolutionary studies (Kuussaari et al. 2000; Hanski and Singer 2001; Hanski 2011) have benefitted from the large amount of ecological and environmental knowledge for the Glanville fritillary system. Recently, genetic studies have expanded to studies of gene expression (Wheat et al. 2011; Kvist et al. 2013). Following the pioneering study on the transcriptome of the Glanville fritillary (Vera et al. 2008b), a manuscript describing the full genome is currently in preparation. The Glanville fritillary project is a prime example of the synergistic research opportunities that often exist in the context of long-term monitoring studies.

Finally, the detailed large-scale mapping of the habitat for the Glanville fritillary has offered unique research opportunities to develop large-scale research projects on other organisms that use the same habitat patch network. Thus, Saskya van Nouhuys and her students and collaborators have worked for more than a decade on the parasitoids of the Glanville fritillary, which has become one of the best known insect metacommunity (van Nouhuys and Hanski 2005; van Nouhuys and Kraft 2012). Anna-Liisa Laine and her students and collaborators have worked for a decade on the coevolutionary spatial dynamics between *P. lanceolata*, one of the host plants of the Glanville fritillary, and the specialist powdery mildew fungus *Podosphaera plantaginis* (Laine and Hanski 2006; Tollenaere et al. 2012). Marko Nieminen has studied the metacommunity of two species of weevils feeding on *P. lanceolata* and their parasitoids (Nieminen et al. 2004; Vikberg and Nieminen 2012).

### Sample and data management

Long-term and large-scale population studies run, sooner or later, into problems with data management – unless data management is taken seriously from the very beginning. The Glanville fritillary project was started, as many comparable projects are, with spread sheets and a simple data base. Over time, when the amount and complexity of the data increased, it became evident that a more sophisticated way of managing data is necessary. Unfortunately, there are no simple solutions for ecology and population biology projects, which often involve complex environmental, demographic, and genetic data; which typically involve spatially referenced data; data originating from observational studies and experiments; and samples for which multiple types of data are obtained, including demographic, phenotypic, and genotypic data.

In the Glanville fritillary project, the basic record is represented by a family group of larvae recorded in a particular population (habitat patch) in a particular year. The group of larvae is given an ID from a running list, and the corresponding printed barcode is used to label, in the field, a tube into which three larvae from the group are sampled. Subsequently, this label is physically moved to a rearing container when the larvae are reared individually, following the winter diapause in the laboratory, and a range of phenotypic traits are recorded. A sample of larvae are reared into the adult stage and a large number of traits related to behavior, mating, reproduction, and longevity are recorded in a large outdoor population cage (Hanski et al. 2006; Saastamoinen 2007, 2008). The larva or adult butterfly is preserved, and the sample enters a pipeline of DNA extraction and genotyping. The EarthCape database platform is used to record all the data related to rearing and phenotypic measurements, whereas genotype data starting from DNA extraction onwards are managed with a dedicated platform for maintaining genetic pedigrees (Progeny Lab, Progeny Software LLC, Delray Beach, FL). All environmental data, which are linked to the records of larval groups via the ID of the habitat patch, are managed with EarthCape. Using the map-based interface in EarthCape, data can be viewed by clicking the respective habitat patch. The spatial coordinates of the habitat patches and even individual larval groups can be viewed on, for example, Google Earth (see Appendix). The Glanville fritillary project highlights a number of features that are essential or at least very helpful while managing large, spatially referenced, and complex data from long-term population studies, and which are run in parallel with experimental studies on the same study system.

## Figures and Tables

**Figure A1:**
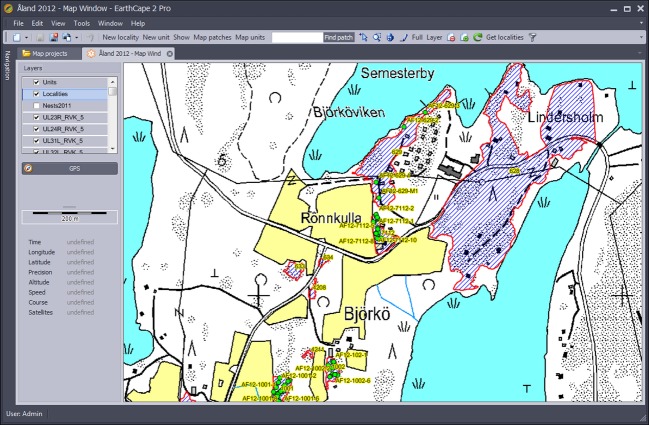
The default screen of EarthCape as seen by field assistants to initiate different actions (described in the text).

**Figure A2:**
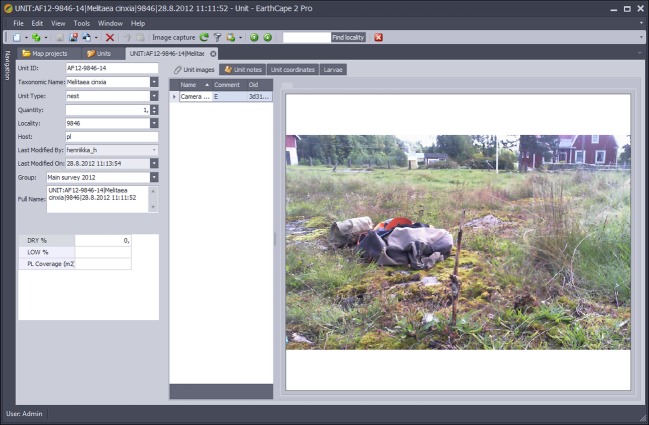
Screen showing information for a selected locality or unit including a picture preview window.

**Figure A3:**
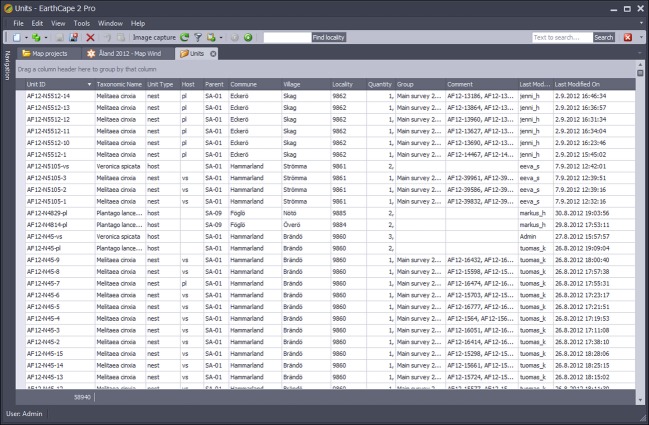
A menu to view localities/units in a list view that can be filtered or grouped in any way the user wants.

**Figure A4:**
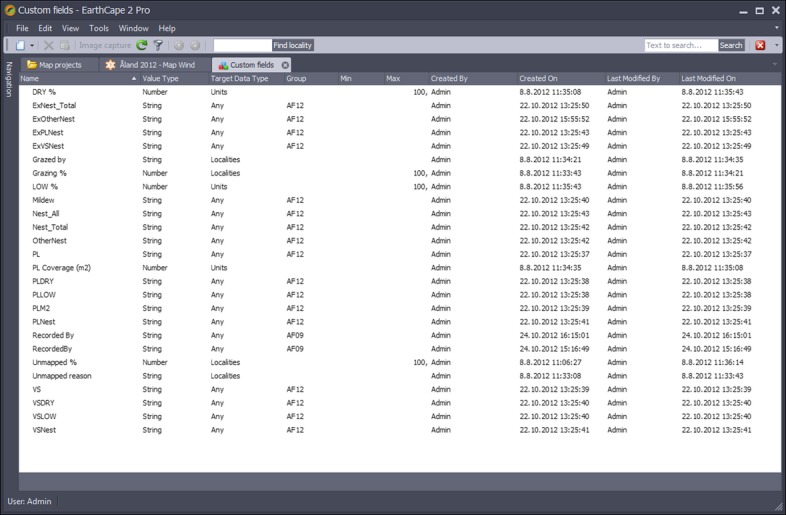
A view of custom fields used in the survey. These fields can be defined per project and can contain all types of data (number, text, binary data, etc.).

**Figure A5:**
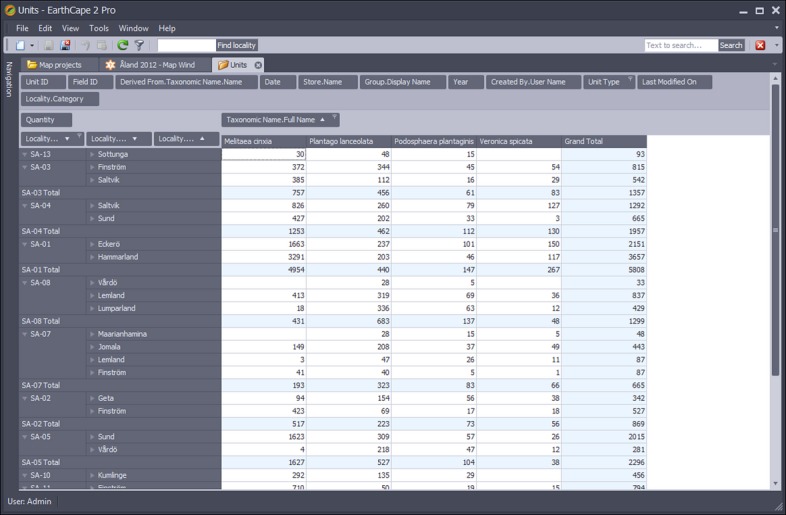
Functions to analyze the data in pivot tables.

## Appendix

### A Brief Description of the Functions of the EarthCape Biodiversity Database

EarthCape is a biodiversity information platform that allows the management of diverse data. The main data components are units (samples/observations), taxonomy, geography, references, genetic data, and custom data. Apart from specific functionalities there are larger domain-specific functional modules: GIS (built-in feature-rich geographic information system), collection management, analysis (pivot table creation, charting), reporting (including label designer), export/import, user interface customization, and field mapping (supports GPS device connection). EarthCape program has been developed by Evgeniy Meyke, who has worked for many years in the Glanville fritillary project. The program has recently become commercially available. Experience gained during the surveys has influenced the development of many components of EarthCape. Before the survey, the field assistants (FA) attend a short demonstration of how to use the program and they are given a short two-page manual summarizing the main functions that they need during the field work.

The EarthCape platform consists of database back-end of the choice of the user, Windows desktop client application, and browser-based web application. In the application for the Glanville fritillary survey, the project coordinator customizes the user interface using built-in capabilities by removing unneeded elements, renaming existing ones to have more relevance for the application, and rearranging the input forms and command layouts. Survey-specific patch parameters are stored in the form of custom data. Each habitat patch is stored as a geographic object. Patch geometry is stored in OpenGIS Well Known Text format as polygons and is fully editable using the built-in GIS editor. Thus, FA are able to make corrections to patch outlines in the field if necessary.

Each pair of FA has a notebook computer with the EarthCape Client installed along with a database containing all the habitat patch information and preloaded records for larval family groups to be recorded (unused records are discarded). Furthermore, each preloaded record for larval groups has three larval records attached to them, corresponding to the set of three larvae to be sampled from each larval family group. Bar-coded labels are printed out and distributed to the FA to be used while sampling larvae. All these arrangements are made to reduce the amount of work in the field. What remains to be done in the field is to find the larval groups, to mark them in EarthCape (GPS assisted), to sample the larvae and place them in an Eppendorf tube, to take a barcode sticker, and to record the barcode into the client program. In this process, both larval groups and individual larval records are geotagged and stored in the database.

The main input methods used in the field work are map based. Åland base maps of different resolutions have been preloaded in the GIS view and their display is dependent on the zoom level. The current location is displayed on the map from a Bluetooth-connected GPS device. The user can either use the coordinates coming from GPS device or double click on the map to record, for example, the location of the larval family group.

Import/export functionality in EarthCape is used to merge the data from each field computer into a master database every evening, which allows the survey director to follow the progress of the field work and to plan future tasks. Checking for errors and general cleanup of the data are performed when the data are merged. The final version of the database following the field work is merged to the main database.

### Brief Guide to the EarthCape Functions Used in the Field

Figure A1 shows the default screen of EarthCape as seen by the FA to initiate different actions. The layout is modified separately (by the survey administrator) for the spring and fall surveys to make it as easy as possible to record the relevant data. In the layout used for fall survey, most of the screen is occupied by the map window, which consists of the background map and overlayed layers for localities (habitat patches) and units (larval groups and other objects). On the left of the map is a menu of the layers available for the current view, the scale bar, and information from the GPS device. Basic functions and wizards that can be started from this screen are shortly described below.

Tools in the top row from left to right

- Create new locality (habitat patch) or unit (larval family group), a wizard that helps in the creation of the process.
- Show information for the selected locality or unit (Fig. A2).
- Show the localities in the current view.
- Show the units (and other marked objects) in the current view.
- Pan and zoom to the selected locality by entering the patch ID.
- Select an object on the map.
- Zoom in/out the map (free level).
- Pan the map.
- Edit the boundaries of the selected locality or edit the location or boundaries of the selected unit (a unit can be delimited either as a point, line, or area or without any spatial information).
- Zoom to full map or zoom to a selected layer (e.g., habitat patch).
- Remove or add map layers with spatially referenced information (e.g., larval family groups found in the previous year or modified habitat patch boundaries).
- Get the list of localities visible in the current view.
- Filter the data according to a specific project (not used in the survey).

Tools elsewhere in the screen and in menus (most commonly used)

- Start/stop reading the GPS signal and show the current location on top of the background map.
- Take a picture (with internal/external web camera) and attach it to a selected object. A link to the picture is stored into database and the picture file to specific data folder.
- Add a note(s) to any object.
- Through a menu the user can also view localities/units in a list view that can be filtered any way the user wants (e.g., by survey area/commune/village/date/last modified by user X, etc.) (Fig. A3).
- Export current map with all the visible localities and units to image (a snapshot that can be used, e.g., to help explaining problematic situation to the survey director, also commonly used to visualize data in presentations).

The administrator of the project can use the program to perform various tasks

- Export and import data between the local database copies in field computers.
- Import additional data from a broadsheet program (Microsoft Excel).
- Create taxonomy, add species, genus, and higher taxa that are needed in the survey.
- Maintain and create custom data fields used in the survey. Custom data fields are user-created data fields that are connected to localities and/or units and are specific to the current project. User can define, for example, the name and type of the field (text, number, binary, list, etc.) (Fig. A4).
- Show data in a third-party GIS program (ArcGIS, Google Earth) by exporting the data to suitable format. A conversion of coordinates between different coordinate systems can be done with a tool included in the program for this purpose.
- Edit the layout, data propertie,s and views of the program (mostly used before the survey).
- Create users and modify their roles (user rights) in the project.
- Analyze the data in pivot tables (Fig. A5).
- Create a report with selected data. Barcodes are printed before the survey using this functionality.

The administrator obtains backups from individual local copies of the database and combines them to a master database, which is transferred to the central database following the field survey. This allows usage of the data by other members of the research group in the office and in the laboratory.

### Description of the Study Landscape

#### Definition of habitat

The larval host plants *P. lanceolata* and *V. spicata* grow on dry meadows, pastures, and comparable habitats, which occur mostly as well-defined, discrete patches in the Åland Islands (Fig. 2A, B). The key criterion of breeding habitat is the presence of at least one of the two host plant species. There is, however, the complication that one has to decide how many and how closely situated host plant individuals have to be within a potential breeding site (meadow) to allow successful reproduction by the butterfly. The larvae feed gregariously in groups that have initially 50–250 larvae (Kuussaari 1998; Kuussaari et al. 2004). Individual host plants are so small that a large larval group will defoliate the entire plant individual on which the female oviposited the egg cluster, and hence the entire larval group has to move to another nearby plant. Therefore, if there are very few host plants and they are very scattered, the site may not allow successful development of even a single larval group, especially in a year during which many plants dried out (below). We have attempted to map all potential habitat patches in our study (below), including the ones which have so little host plants that in unfavorable years there is not enough food for even a single larval group. Such habitat patches comprise <1% of all the meadows that have been mapped.

#### Description of habitat patches

During the annual surveys in late summer, the following characteristics of habitat patches are recorded:

##### Does the habitat patch exist?

The first thing to observe while starting to survey a habitat patch is whether host plants are present at all in that year. It is often difficult to decide whether a meadow should be considered as a habitat patch even if one follows the rules described in the main text. There are two main reasons why a patch may no longer exist: overgrowth, meaning that the larval host plants have lost in competition with other plants, typically grasses; and various construction works. Overgrowth may have become faster in recent decades due to the increasing deposition of atmospheric nitrogen (Dalton and Brand-Hardy 2003). The amount of grazing has decreased for decades in Finland, but has recently increased in the Åland Islands (see Fig. 6 in the main text), which helps keep grazed areas favorable for the Glanville fritillary.

##### Grazing status

Roughly 42% of the habitat patches were grazed in 2011, usually by cattle or sheep but also by horses. Grazing by wild animals is not possible to record, but is probably much less important. Grazing lowers the quality of a patch in the short term (host plants are fed and trampled upon), but improves it in the long run (Hanski et al. 1995). Grazing intensity, the species, and the proportion of grazed area are recorded. Possible mowing of the meadow is also recorded.

##### Unmapped part of a patch

The proportion of a patch that could not be surveyed is estimated, typically when potentially dangerous grazers (bulls, stallions, ostriches) are present. The number of partially or totally unmapped habitat patches is very small (e.g., 25 of 3810 patches in 2011).

##### Abundance and cover of host plants

First, the abundances of the host plants *P. lanceolata* and *V. spicata* are estimated separately using the following abundance categories: 0 = not a single individual present; 1 = very sparse, no dense groups of plants; 2 = at least one dense group of plants, which could support at least one larval group, but no larger numbers of larval groups; and 3 = at least one large high-quality patch of plants that could support tens of larval groups. The distinction between Classes 2 and 3 is somewhat subjective. Second, the areas covered by *P. lanceolata* and *V. spicata* are estimated separately for both species. These areas can subsequently be transformed to percentage coverage. In practice, this measure is very difficult to estimate accurately, mainly because of large variability in host plant density. In our analyses of the data, we have mostly used the abundance categories rather than the area covered by the host plants (Hanski 2011, 2012a).

##### Percentage of desiccated host plants

The percentage of totally desiccated plants/leaves is estimated separately for the two host plant species. Drying out of host plants can seriously increase larval mortality and in extreme cases cause local extinctions.

##### Height of the vegetation surrounding host plants

Both host plants and larvae do best in relatively low vegetation (e.g., postdiapause larvae bask in the spring to increase their body temperature; Kuussaari 1998; Boggs and Nieminen 2004). *P. lanceolata* occasionally grows in relatively tall grass-dominated vegetation, which is typical for overgrown meadows. The parameter recorded is the percentage of individual plants surrounded by low vegetation.

##### Habitats surrounding the patch

The percentage of patch boundary bordering forest, semiopen habitats (e.g., sparse and low trees growing on rocky terrain), cultivated fields, water bodies (ponds, lakes or sea), and other open areas (e.g., pastures, fallows, yards, parking places) was recorded when the habitat patch network was mapped in 1993 and 1998–1999. The presence of roads is recorded, but patch boundary is recorded based on the habitat type beyond the road because the typical, very narrow roads in Åland do not form barriers for butterflies, and host plants often grow on road verges. On the other hand, closed patch boundary (especially forest but also semiopen habitat) very efficiently prevents movements by the Glanville fritillary; see radar-based tracking records in Ovaskainen et al. (2008). Different types of open terrain probably have different resistance to movements, but these effects are not well known (but see Moilanen and Hanski 1998). We do know, however, that overall openness of the patch boundary increases emigration rate (Kuussaari et al. 1996). Therefore, the openness of the patch boundary influences the effective isolation of a particular habitat patch. In later years, information on patch boundaries have not been updated, as the values remain relatively constant. Since 2009, we have started to take photographs of the patches from one or few representative places, decided by the FAs visiting the patch, to be able to verify the status of the patch and the development of its surroundings over the years.

##### Abundance of nectar plants

Abundance of nectar plants was estimated in early years as the proportion of total patch area covered by vegetation dominated by *Alopecurus pratensis* and *Anthriscus sylvestris*. However, due to difficulties in defining the vegetation type in late summer especially in dry years, we have stopped recording this variable. The above-mentioned vegetation type usually includes several plant species that flower abundantly during the flight season in June. Availability of nectar enhances egg production (Boggs and Nieminen 2004), and high abundance of nectar plants decreases emigration rate and increases immigration rate of the Glanville fritillary (Kuussaari et al. 1996).

#### Mapping of the habitat patch network

The initial mapping of the entire study area (Fig. 3) was done in late summer 1993. There are 908 km (in 1995) of paved and unpaved public roads and roughly 2500 km of narrow unpaved farm roads in the Åland Islands. During the mapping, most roads were driven through and all potential sites close to the roads were visited. Using topographic maps, potential habitat located further away from the roads, mostly dry meadows on rocky outcrops, was identified and visited. The original mapping was done by 20 FA (biology students) in 2 weeks working in pairs with a car. This survey yielded 1238 habitat patches. During the annual surveys of butterfly populations in subsequent years (below) new habitat patches were discovered at the rate of roughly 50 patches per year. Entirely new patches are created by grazing and mowing formerly overgrown grassy areas, and less frequently by other forms of disturbance. However, most of the newly discovered patches were not new as such, but merely patches that had remained unnoticed in the initial mapping.

In the summers of 1998 and 1999, the entire Åland Islands was resurveyed very thoroughly by systematically driving through all the roads, including small farm roads. All potentially suitable areas where no habitat patches had previously been delimited were surveyed by foot, which meant walking around most cultivated fields and inhabited areas. Potentially suitable areas further away from roads were located with maps and visited. Furthermore, some larger islands with no road connection were mapped. However, extensive rocky areas with no roads as well as most of the shoreline remained unvisited due to the very high extra effort that would have been needed to cover them completely. On the other hand, most of the rocky areas and shoreline are unsuitable for the Glanville fritillary, and suitable patches in such areas appear to be clustered and only exist in some localities. The resurvey yielded ca. 2900 new patches, thus increasing the total to more than 4200 patches. These figures appear to suggest that the original survey was very inaccurate, but it should be noted that the very thorough second survey aimed at discovering even the smallest patches, patches of very low quality and patches on islands currently unoccupied by the Glanville fritillary. Thus, the pooled area of the patches mapped in 1993 was 338 ha, whereas the pooled area of previously unmapped habitat that was mapped during 1998–1999 was 236 ha. The current number of valid habitat patches in the database is 4248 (in 2012) with the pooled area of 783 ha, which covers ca. 0.5% of the total land area in the Åland Islands (1 552 km^2^). This number of patches includes deletions and merged patches as well as the discovery of new ones in the past decade.

#### Delimitation of individual habitat patches

Patch area is a key parameter, as it has played a critical role in the development of metapopulation models in the context of the Glanville fritillary project (Hanski 1994; Hanski and Ovaskainen 2000; Hanski et al. 1996, 2011; Ovaskainen and Hanski 2004). The basic rule is that one meadow or a comparable site is one discrete habitat patch. This definition follows from the behavior of adult butterflies, which are more or less randomly scattered across a meadow while feeding, searching for mates, and ovipositing, but which generally avoid crossing the boundary of the meadow (Ovaskainen 2004; Ovaskainen et al. 2008). While the entire Åland Islands were resurveyed during 1998–1999, all patches were delimited in the field by only three FAs to make the delimitation as uniform as possible. The patch boundary was walked around with a GPS receiver to record the boundary. During 1998–1999, we used Magellan ProMARK X-CM (Magellan Inc.,) with MSTAR 2.06 software (Magellan Inc., Santa Clara, CA), and real-time correction by YLE Fokus 2-m RDGPS in mobile surveying mode with 3-sec observation interval. From 2000 onwards, we have used CMT Alto G-12 GPS receiver (Corvallis Microtechnology Inc.,) with PC-GPS 3.6 software (Corvallis Microtechnology Inc.). We have also recorded selected fixed points along patch boundaries. The real-time correction has 0.5–2 m accuracy under favorable conditions (Fig. 4). The coordinates of patch boundaries were transferred into a GIS program and combined with electronic maps which greatly facilitates the survey of butterfly populations in the field (below).

The delimitation of habitat patches has been complicated by three factors. First, meadow boundaries are often diffuse, for instance, between a meadow and semiopen rocky area with small trees, or between a meadow and unsuitable tall grass or herb-dominated area without host plants. All tall grass and herb-dominated vegetation and semiopen areas without host plants are excluded from the patch. On the other hand, in many cases, the boundaries are entirely unambiguous, as the meadows are frequently bordered by tall forest, cultivated fields, roads, gardens, and water bodies (Fig. 4).

The second complication is due to other nearby meadows. It is not unusual that two or several discrete habitat patches are located close to each other, but separated by some completely unsuitable habitat, for example, a narrow stretch of cultivated field, a group of trees or a road. Our rule has been that ca. 20 m of nonhabitat and ca. 50 m of otherwise suitable habitat but without host plants separate two patches. By this definition, there are no roads that would divide a patch into two patches, although the area covered by, for example, a road is excluded from the area of a patch. Three exceptions have, however, been recognized. First, on pastures we avoid including often very large areas (several ha) of short turf with no host plants by identifying the parts of pastures with host plants and delimiting them as separate patches. These intervening areas are furthermore very susceptible to overgrowth if grazing is terminated. On the other hand, if host plants occur throughout the pasture, the entire pasture is considered as one habitat patch. Of the current suitable habitat for the Glanville fritillary, 41% is represented by active pastures. Second, very narrow and long corridors of apparently suitable habitat connecting two larger patches of habitat are ignored and two separate patches are delimited instead. Third, relatively small rocky outcrops (suitable habitat) completely surrounded by a cultivated field separated by less than 20 m from other suitable habitat are mostly considered separate patches. Finally, some patches have been merged in each year, for instance, when host plants have spread between two or more patches due to, for example, resumed grazing, thus creating an area that satisfies the definition of a single patch. The abundances of both host plant species can vary considerably from 1 year to another and therefore a particular habitat patch may have very little host plants in a year but a high density in another. In the annual survey, host abundance is one of the patch variables that are recorded (below).

The third main complication in the delimitation of habitat patches is due to sporadic occurrence of the host plants, especially *P. lanceolata*, at sites that are otherwise not considered as habitat patches. For example, one has to decide whether narrow road verges with some host plants are patches or not. In practice, we have only included road verges where host plants are abundant or where the verge, with lower host plant density, is directly connected to suitable habitat, in which case the habitat patch and the road verge together comprise a single habitat patch.

### Metapopulation Survey

#### The logistics of the survey

The annual metapopulation survey is organized in late August to early September, at the time when the larvae have woven a winter nest that is relatively easy to find in the field (Fig. 2C). The number of habitat patches visited in each autumn has varied somewhat from year to year depending on the exact number of habitat patches known at the time, but also because in many years habitat patches on smaller islands that have been unoccupied in the recent past have not been surveyed to reduce costs. In the autumn 2011, the number of patches surveyed was 3808.

The FA are mainly undergraduate biology students and therefore it is easy to explain to them the rational of the survey and the field methods. In the years 1993–1997, ca. 18 FAs were employed, but the number was increased to ca. 35 following the resurvey of habitat patches in 1998–1999. Since 2009, the intensity of the survey was further enhanced to increase the probability of detection of larval groups, and the number of FAs was doubled to ca. 70. To allow comparisons with the results for the previous years, the search of larval groups was now divided into two phases. In the first phase, FAs followed the original search procedure, search times, and related parameters (below). In the second phase, a selected set of patches were completely re-searched by the same FAs or by another group of FAs (depending on the year), and the larval groups detected during this second phase were recorded separately into the database. Roughly one third of all patches have been re-searched in this manner since 2009.

The field work is done by pairs of FAs. Whenever possible, at least one FA in the pair has previous field experience. Prior to the survey, the FAs attend an orientation lecture, and survey-related concepts are demonstrated in practice in the first day in the field, including finding of larval groups and recording of habitat patch and host plant-related parameters. Each group of FAs has a car, and they end up driving 50–100 km per day while visiting and surveying on average 20 habitat patches per day. Most of the land is privately owned, but the land owners have almost always reacted positively toward FAs and have even been interested in the work done on their land. It has not been possible to contact the vast majority of the land owners individually before the surveys, but FAs are asked to explain the basics of the work to anyone interested in. The FAs have a one-page description of the long-term study to hand over, and we frequently have articles about the research in local newspapers, making the project familiar to many locals.

The habitat patches have been divided into six size categories, namely, the smallest patches down to 10 m^2^, <500, <2000, <10,000, <20,000 m^2^, and the largest patches up to 10 ha. Each size category has a target search time, which the FAs use while searching for larval groups in the patch. The time varies between 10 and 60 mins for the first five categories (Table A1). In special occasions, for instance, in the case of the largest habitat patches or with very high density of host plants, these target times can be exceeded and maximally doubled. In the given time, the FAs walk through the entire patch area and examine closely all host plant clumps. If no larval groups are detected, the entire patch is re-searched using the same target time, to make as certain as possible that the particular habitat patch was unoccupied (this is important for patch occupancy metapopulation models; [Ovaskainen and Hanski 2004]). However, if at least one larval group was found during the original search time, there is no re-search. The time used to enter the data in the database, to mark the larval groups (with a small wooden stick in the field and with spatial coordinates in the database), and sampling of larvae (below) are excluded from the search time.

**Table A1:** The approximate minimum search times in one meadow for a pair of field assistants

Patch size (m^2^)	Search time (min)
<500	5
500–2,000	10
2,000–10,000	15
10,000–20,000	20
20,000–50,000	30
>50,000	Enough to cover the entire area

The search time is specified for six different size classes of meadows.

FAs are encouraged to create new habitat patches in the database, if they find an area fulfilling the criteria of a habitat patch. The number of new patches created each year has varied from a few to almost 50, much depending on the weather conditions preceding the survey, as fresh host plants are more easily detected than dried ones. There are also some tens of patch deletions and merges every year; these modifications are suggested by FAs and approved by the person supervising the survey. We try to avoid overaggressive modification in the patch network, but unambiguous changes are recorded.

In the spring, the habitat patches in which larvae were found in the previous autumn are searched for postdiapause larvae (Fig. 2D). Timing of the spring survey is critical, as the larvae are easier to detect when they have had time to grow since breaking the diapause in late March or early April. At the same time, the larvae should not yet have reached the final instar because at this stage they start to disperse and it is no longer possible to count them accurately. The ideal time varies from a year to another but is usually at the end of April. At this stage, the larval groups can be found relatively easily with the help of the spatial coordinates and photographs of the nest surroundings recorded in the laptop in the previous autumn as well as with the help of the small wooden sticks marking larval groups in the field (above). The same parameters related to larval groups are recorded as in the autumn, but now additionally the groups that disappeared during the winter and possibly previously undiscovered groups are recorded, and the number of individual larvae in each group is counted. In the spring 2012, which was a record year (Fig. 6), 137,000 larvae were thus counted. Additionally, the numbers of hatched and nonhatched cocoons of the primary parasitoid *Cotesia melitaearum* (Wilkinson) are counted for each larval group (for description of the parasitoid assemblage, see Lei and Hanski 1997; Lei et al. 1997; van Nouhuys and Hanski 2004).

#### The cost of the survey

In 2010, the total cost of the autumn survey was around €150,000. The spring survey is cheaper, typically €15,000–20,000, because only the populations found in the previous autumn are visited. The FAs are paid on an hourly basis according to the University salary scale. In addition, the FAs are offered student credits for participating in the field work and attending a series of lectures on the survey and related research. Salaries account for around 75% of the total cost of the survey. Other major items include travel expenses to and from the study area and during the field work and accommodation during the survey. Accommodation has been provided in rented cabins (summer cottages) with basic facilities. Use of the employees' own cars is required and compensated for as travel allowance (0.46 €/km in 2010). All other travel-related expenses are reimbursed, including the costs of the ferry from the mainland to the Åland Islands and smaller ferries operating between the islands within the survey area. Despite the large number of devices needed in the survey, including computers, extra batteries, car chargers, GPS devices, and so forth, one-time cost of obtaining them is about €15,000, only 10% of the yearly cost of the survey. Furthermore, the equipment can mostly be used for several years and the yearly costs consist only of repairs and the purchase of new devices to replace the ones that do not function properly, a sum that has been only some hundreds of euros yearly.

#### Recording the data in the field

Recording of the data in the field has been under constant changes since 1993 due to development in computer hardware. Since 2010, all data have been recorded directly on small rain-protected notebook computers, which contain a local copy of the master database. The computers have the topographic map of the Åland Islands to help the FAs orient themselves to the next habitat patch. When on the spot, they may zoom in to the patch level to show the patch outline displayed in the GIS viewer on top of a detailed topographic map. All other patch-specific information is also available, such as the records of larval groups, past information on host plants, and so forth. The survey coordinator collects the data from each computer into a single database every evening to construct preliminary pivot tables that enable spotting missing data, obvious outliers, areas still to be surveyed, and so forth. The ability to see and explore the data on the map and the easy preview of the data in Google Earth make a big difference when cleaning up the data. Data are synchronized using EarthCape import/export mechanism, which also serves a backup purpose. The merged data set can next be redistributed back to the field computers for the following day, which helps the surveyors in case the computers are reshuffled or used for tasks that require the data from the previous days to be available in, for example, the surveys of larval parasitoids and a host plant pathogen (below).

Turning to the data recorded during the survey, the most important data concern the larval groups. When a new larval group has been detected, the FAs will mark its spatial location simply by clicking on the map or by recording from the GPS receiver. Some manual adjustment of the spatial location is often needed, as even in the open habitat where the majority of the habitat patches are located the accuracy of the regular GPS signal is limited to several meters, and it is not uncommon to find several larval groups within the margin of error. Additionally, the larval group is marked physically with a small inconspicuous wooden stick in the field. The coordinates thus recorded guide the FAs to the vicinity of the larval group during the spring survey (above), where they should be able to see the stick, the remains of the winter nest, or basking larvae. In recent years, a photograph has been taken of the larval web and its surroundings with a camera integrated into the notebook or with a separate web camera (Microsoft LifeCam HD-6000, Microsoft Corp., Redmond, WA). The purpose of these photographs is to provide additional help to the FAs in the spring, an option that has proven to be helpful.

The database has template records for new larval groups, which are employed in the recording. The FAs need only to enter the next barcode information for the new larval group, thereby making sure that the same label (barcode) is associated with the sample of larvae taken from the group (below). Ideally, one would use barcode reader for the purpose, but so far the code has been entered manually. In addition to the spatial location of the larval group, the host plant species on which the group was found is recorded in the database. The vast majority of larvae feed on *P. lanceolata* and *V. spicata*, but a few larval groups have been found on other congeneric plant species over the years (*P. major, P. media, P. maritima, V. chamaedrys, V. longifolia, V. officinalis,* and *V. serpyllifolia*; [Kuussaari et al. 2004]). Occasionally larval groups split into two or even three “split-groups”, and conversely two or more groups may merge. The split groups usually occur within less than half a meter from each other. For simplicity, possible split groups are, however, counted as separate groups. Finally, several parameters of the habitat patch are recorded, including whether the patch actually exists (host plants present), grazing status, abundance and cover of host plants, percentage of desiccated host plants, and so forth. These variables are described in detail above.

#### Weather data

Weather conditions play an important role in the dynamics of the Glanville fritillary metapopulation (Nieminen et al. 2004; Hanski and Meyke 2005). Precipitation data have been obtained from weather radar since 1998. The spatial and temporal resolutions of these data are 0.5 × 0.5 km and 5 min, respectively. In addition, we have placed portable temperature and humidity data loggers (Lascar Electronics, EL-USB2, Salisbury, U.K.) in 50 representative patches starting in 2009. The loggers are placed in the field in early April and recovered during the fall survey during August to September. The loggers are mounted about 30 cm above the ground and shaded from direct sunlight with a white plastic half-dome cover. A separate black button recorder (Maxim iButton DS1922L, Sunnuvale, CA) is planted in a subset of the sites to measure temperatures that basking larvae are able to reach in the spring.

#### Sampling of populations

In 1995, 2002, and every year since 2007, a sample of two or three larvae has been taken from every larval group detected in the field for phenotypic and genotypic measurements. A small hole is made to the bottom part of the winter nest with forceps, and the larvae sampled are placed into a 2-mL Eppendorf tube with a piece of cotton wool to absorb extra moisture and to prevent larvae from becoming moldy. The lid is punctured with the tip of tweezers to allow air circulation in the tube. Due care is taken to keep the level of disturbance as low as possible. At the time of the survey, the larvae are still active and repair the hole in the nest by weaving new thread. Information on the larval sample is entered into the database at the time of sampling. The tubes with larvae are labeled with preprinted barcode labels with appropriate information and stored in a cool dark place until transferred to the laboratory. Labels are read into the database in the field, and the same codes are used subsequently while rearing the larvae and taking phenotypic measurements as well as for DNA samples. Using barcodes reduces errors in the labeling of samples, and reading barcodes saves time, which is an important consideration when dealing with thousands of samples.

#### Reliability of the survey

Given the size of the study area (50 by 70 km) and the number of discrete habitat patches (ca. 4000), it is obvious that the survey of population sizes cannot be exhaustive. Several approaches have been used to estimate the probability of detecting a larval group during the autumn surveys. In 1994, 1995, and 1997, intensive surveys of four habitat patches (different patches in each year) were conducted to obtain a value for the “true” number of larval groups, after which eight independent pairs of FAs conducted the survey with the usual search effort (Table A2). In 2008, 67 patches were surveyed twice, with the same search effort in each survey, this time the second pair of FAs knowing the nest count from the first survey. Using a Bayesian model to analyze these data sets, Harrison et al. (2011) estimated that 50% of the “true” number of larval groups was found during the first search. In 2009 and 2011, a group of habitat patches (180 and 80, respectively) were thoroughly resurveyed by a large number of FAs. Altogether, 1304 larval groups were found in these patches, 809 (62%) of which had been detected already during the first search. Assuming that almost all larval groups were found during the thorough re-search, we can conclude that the detection probability for a larval group is 0.5–0.6 in the regular survey.

**Table A2:** Summary of the results on the estimated numbers of larval groups in control patches

Year		Patch I	Patch II	Patch III	Patch IV	Total no. of groups	Mean % found
1994	Mean	1.1	6.5	1.3	5.5	28	51.4
	Range	0–2 (3)	5–10 (13)	0–3 (4)	3–8 (8)		
1995	Mean	1.5	6.4	5.4	0.1	28	50.7
	Range	0–3 (3)	4–8 (9)	2–8 (15)	0–1 (1)		
1997	Mean	2	0.8	2.4	0	11	44.2
	Range	0–4 (4)	0–2 (2)	1–5 (5)	0 (0)		

The total number of larval groups detected in each patch is given in parentheses. The patches that were used as controls were different in each year.

When the patch is judged to be unoccupied during the first search, it is immediately re-searched with the same effort as in the first search. In the controls done in 2009 and 2011, 72% of the patches considered to be unoccupied turned out to be unoccupied also after the second search. Of the remaining 28%, half had only one or two larval groups and the rest had >2 larval groups based on the second search. The most common reason for missing larger numbers of larval groups in the first search was that a part of the patch had not been searched at all for some reason, for instance, because the patch boundaries were misinterpreted. This problem has been largely eliminated in recent years by having the outline of the patch displayed on an accurate topographic map in the field computer. In years when no systematic controls have been done, the survey efficiency has been controlled in a less systematic fashion. For instance, control visits by the survey coordinator or a group of experienced FAs have provided feedback to original surveyors to maintain high motivation and to instruct the less experienced FAs.

### Field Assistants

We acknowledge the contributions of the following FA who have participated in the long-term monitoring of the Glanville fritillary metapopulation during 1993–2012.

**Table:**

Aino Ahvo	Heikki Savolainen	Laura Nummelin	Riitta Toivonen
Aino Hosia	Heikki Torkkeli	Laura Nurmi	Rosemary Setchfield
Aino Tyyskä	Heini Karvinen	Lauri Kajander	Saana Kemppi
Aki Aintila	Heini Lankia	Lauri Sillantie	Saara Jussila
Aki Saarela	Helena Antikainen	Lea Heikkinen	Saara Karjalainen
Aki Suzuki	Helena Koivulehto	Leena Jyrinki	Saara Kilpinen
Aleksi Lehikoinen	Helena Rosenlew	Leena Laitala	Saara Tähtinen
Aleksi Mäntylä	Heli Routti	Liisa Maanavilja	Saara Vauramo
Aliisa Harjuniemi	Henna Makkonen	Lina Munsterhjelm	Saila Kuokkanen
Alma Oksanen	Henri Järvisalo	Linda Peltola	Salla Heiman
Anastasia Labudina	Henriikka Hallamaa	Linda Rosqvist	Salla Rantala
Anita Etholén	Heta Rousi	Maija Ihantola	Salla Sacklen
Anna Backholm	Iida Autio	Maiju Lanki	Salla Suomensaari
Anna Hahtola	Ilik Saccheri	Malin Ek	Sami Lindgren
Anna Haukka	Ilona Merikanto	Malla Kirjokangas	Sami Ojanen
Anna Hietala	Ilona Oksanen	Mar Cabeza-Jaimejuan	Sampsa Vilhunen
Anna Talvitie	Ina Naukkarinen	Mari Kekkonen	Sanja Hakala
Anna Weckman	Inkeri Sole	Mari Koskela	Sanna Eronen
Anna-Kaisa Kähkölä	Irina Sytcheva	Mari Paakkinen	Sanna Kokkonen
Anna-Leena Mäkelä	Jaana Komonen-Kauramäki	Mari Pihlajaniemi	Sanna Korkonen
Anne Halmepuro	Jan Salonen	Maria Degerlund	Sanna Leppänen
Anne Hirvonen	Janette Baarman	Maria Tuomi	Sanna Mäkeläinen
Anne Holma	Janna Hemming	Marianne Kovasin	Sanna Pekuri
Anne Koivunen	Janne Granroth	Marika Tikka	Sanna Suikkanen
Anne Luoma	Janne Hakanen	Marja Kosme	Sanna-Riikka Remes
Anne Lyytinen	Janne Heliölä	Marjo Kuronen	Sanni Monto
Anne-Marie Hietala	Jan-Peter Bäckman	Marjo Mansikkamäki	Sanni Turunen
Anni Arponen	Jari Ilmonen	Marjo Saastamoinen	Sara Neggazi
Anni Joensuu	Jari Julkunen	Mark Camara	Sari Haikola
Anniina Mikkonen	Jarno Tuimala	Markku Lahtinen	Sari Suhonen
Annika Koskinen	Jasmina Uusitalo	Markku Ojanen	Saskya van Nouhuys
Annika Vartio	Jenni Hämäläinen	Marko Nieminen	Satu Laitinen
Anni-Mari Pulkkinen	Jenni Jäänheimo	Marko Pesu	Satu Viitasalo
Ann-Marie Antaon	Jenni Korhonen	Markus Haveri	Senja Piiroinen
Annu Tertsonen	Jenni Lehtimäki	Markus Seppälä	Siiri Fuchs
Anou Londesborough	Jenni Leppänen	Markus Tietäväinen	Sini Mursinoff
Anttu Lipponen	Jenni Punttila	Matti Koivula	Sini Penttilä
Anu Hjelt	Jere Salminen	Meeri Mäki	Sini Wallin
Aura Raulo	Johan Ekroos	Mervi Laitinen	Sirke Piirainen
Auri Koskenmäki	Johanna Ehrnsten	Mia Kraft	Sofia Gripenberg
Camilla Ilmoni	Johanna Eklund	Mia Vaittinen	Sofia Virtanen
Carina Järvinen	Johanna Pelkonen	Miina Fagerlund	Soili Huttunen
Catherine Henricson	Joni Uusitalo	Mika Kalliovirta	Steve Matter
Christina Church	Jonna Katajisto	Mika Saarenpää	Susanna Airaksinen
Coong Lo	Jonne Linna-Alho	Mika Siljander	Susanna Halttunen
Eeva Huitu	Jouko Pokela	Mikko Heini	Susanna Heiman
Eeva Punju	Juha Lumme	Mikko Kolkkala	Susu Rytteri
Eeva Putro	Juha Pöyry	Mikko Kuussaari	Suvi Ikonen
Eeva Saarinen	Juhani Sirkiä	Mikko Putkonen	Tapio Gustafsson
Eevi Rissanen	Jukka Alasaari	Mikko Sonninen	Tarja Kainlauri
Eija-Leena Laiho	Jukka Hari	Mikko Tiira	Terhi Lahtinen
Elina Karhu	Jukka Ikonen	Milja Aitolehti	Terhimarja Malkavaara
Elina Nystedt	Jukka Rintala	Milla Vahtila	Tero Kirjosalo
Elina Uotila	Jukka T. Lehtonen	Minna Brunfeldt	Tero Lukkari
Elisa Metsovuori	Jussi Ikävalko	Minna Lyytikäinen	Tiia Reinikainen
Elli Lappalainen	Jussi Mäkinen	Minna Pekkonen	Tiina Laakkonen
Elli Mattila	Jussi Nygren	Minttu Hannonen	Tiina Pasanen
Emmi Kytölä	Jyrki Holopainen	Mira Korpi	Tiina Riikonen
Emmi Vähä	Kaisa Kantanen	Mirjami Smalén	Timo Pakkala
Essi Ahopelto	Kaisa Luodekari	Netta Lempiäinen	Timo Tuuri
Eva Ehrnsten	Kaisa Rämö	Niclas Fritzén	Tinto Aaltonen
Eva Jansson	Kaisa Torppa	Niklas Kumlin	Tobias Tamelander
Eveliina Kallioniemi	Kaisa Torri	Niklas Wahlberg	Tommi Tolvanen
Eveliina Turpeinen	Kaisu Mankinen	Nina Rautjärvi	Toni Cases
Eveliina Tuurnas	Kalevi Trontti	Nina Siitonen	Topi Lehtonen
Frank Hering	Kalle Huttunen	Ninni Mikkonen	Tuomas Kankaanpää
Guangchun Lei	Kalle Saramo	Noora Hildén	Tuomas Uola
Hanna Aho	Karolina Stenfors	Nora Arnkil	Tuomo Pihlaja
Hanna Kauko	Kata-Riina Valosaari	Nuutti Kangas	Tuukka Virtanen
Hanna Koivula	Kati Yläranta	Olli Ojala	Tuulikki Seppälä
Hanna Koskinen	Katja Bargum	Otso Häärä	Ulla Tuomainen
Hanna Laakkonen	Katja Bonnevier	Outi Lerssi	Unni Pulliainen
Hanna Parkkonen	Katja Enberg	Outi Pihlajamäki	Varpu Pärssinen
Hanna Parri	Katja Fedrowitz	Outi Viinikka	Vaula Lukkarinen
Hanna Paulomäki	Katja Ojala	Paavo Hellstedt	Veera Kuparinen
Hanna Seitapuro	Katja Rönkä	Panu Hälvä	Veera Tuovinen
Hanna Taavitsainen	Katri Korpela	Pasi Ala-Opas	Veli-Pekka Antti-Poika
Hanna Uusimaa	Katri Luukkonen	Pasi Partanen	Venla Kontiokari
Hanna Wahlberg	Kiira Nordqvist	Patrik Karell	Vesa Hyyryläinen
Hanna-Leena Henttinen	Kim Jaatinen	Paula Peitola	Vesa Selonen
Hanne Rajanen	Kirsi Järvinen	Paula Rantanen	Ville Vepsäläinen
Hanne Rapeli	Kirsi Reponen	Paula Uotila	Vilppu Välimäki
Hanne Vihervaara	Krista Viren	Peetri Pihko	Virpi Immonen
Hannele Penson	Kristian Lindqvist	Pekka Saarinen	Virpi Lintunen
Hannele Pihlajamäki	Kristiina Hildén	Peter Kullberg	Virve Koivuranta
Heidi Björklund	Kristiina Visakorpi	Petra Lattunen	Wee Tek Tay
Heidi Hautala	Kristjan Niitepõld	Petteri Mönkkönen	Zdravko Kolev
Heidi Kohtala	Kukka Kyrö	Pia Välitalo	Zeren Gürkan
Heidi Viljanen	Lasse Lindqvist	Reetta Ljungberg	
Heikki Ajosenpää	Laura Hiisivuori	Riikka Kaartinen	
Heikki Koponen	Laura Jäättelä	Riitta Ovaska	

## Supporting Information

**Video S1.** An introductory video of field survey (2:02 min).

**Video S2.** A video showing field survey data visualized with Google Earth (1:39 min).
